# Ad hoc surveys at the Robert Koch Institute

**DOI:** 10.17886/RKI-GBE-2018-088

**Published:** 2018-09-19

**Authors:** Patrick Schmich, Johannes Lemcke, Marie-Luise Zeisler, Anja Müller, Jennifer Allen, Matthias Wetzstein

**Affiliations:** Robert Koch Institute, Berlin, Department of Epidemiology and Health Monitoring

**Keywords:** TELEPHONE INTERVIEW, METHODOLOGIES, HEALTH MONITORING, QUALITY ASSURANCE, PROJECT MANAGEMENT

## Abstract

The Robert Koch Institute (RKI) regularly conducts nationally representative cross-sectional studies (KiGGS, DEGS and GEDA) as part of the nationwide health monitoring system. In addition to these health surveys, data is collected in telephone interviews either on specific thematic fields (such as diabetes) or specific groups (such as medical staff) that were not or only insufficiently covered by the larger health surveys. As they are flexible and fast, ad hoc surveys conducted via telephone interviews can respond to specific epidemiological and health political questions. This article describes the procedures applied in ad hoc telephone interview surveys, which were newly introduced as a standardised method in 2017 and are applied by the Laboratory for Health Surveys at the RKI. The article presents the stages of project management such as concept development, establishment of a concept for data protection, questionnaire development, pre-test and field phase, calculation of weighting factors and provision of the final data set. The aim is to describe the process and shed light on the standardised procedures, the reported quality indicators and the breadth of possible scenarios of application.

## 1. Introduction

The Robert Koch Institute (RKI) regularly conducts nationally representative cross-sectional studies as part of the nationwide health monitoring system. These surveys include the German Health Interview and Examination Survey for Children and Adolescents (KiGGS) [1-3] as well as the German Health Interview and Examination Survey for Adults (DEGS) [4]. As regards their content, the broad spectrum of the data collected as well as preparation and implementation of data collection render them highly complex surveys requiring lengthy preparatory work. For example, sampling for these surveys through the official population registries is highly time-consuming and therefore an additional factor to consider. The planning, implementation and preparation of such extensive surveys is usually a process which takes several years. Often, however, new questions arise that cannot be mapped out by these large surveys or only in an insufficient way. This creates the need for additional telephone interview surveys on specific issues (such as diabetes [5]) or for interviews with particular groups of people (such as physicians). Flexible and most importantly fast, these ad hoc surveys provide information that complements examination and interview surveys such as KiGGS and DEGS and on the short-term produce information on health-related issues.

Ad hoc research is not a new approach at the RKI. Between 2008 and 2014, in addition to KiGGS and DEGS, the German Health Update (GEDA) regularly conducted health interviews [6-9]. From 2008 to 2010, GEDA telephone interview surveys took place in-house, and were outsourced 2012 for the first time.

The need to rapidly conduct surveys at the RKI has increased continuously and as maintaining a permanent and efficient in-house Call Center is not possible, an external market and social research institute (USUMA GmbH) was commissioned in 2017, initially for a period of four years. The contracted services include conducting pre-tests and telephone interviews, quality assurance, data processing, provision of sets of data for analysis including weighting factors, as well as report compilation. Despite outsourcing the data collection, the RKI maintains significant influence and monitors planning, quality assurance and supervision as well as the training of interviewers. Table 1 provides an overview of the ad hoc surveys that have been conducted since 2017.

## 2. The course of the ad hoc surveys

At the early stage of the projects, the Laboratory for Health Surveys (LfG) assists USUMA GmbH (the company contracted by the RKI) in developing a concept for data collection and thereby serves as a coordinating body and intermediary point of contact. While project leaders are responsible for the conceptual development of questionnaires, they may receive methodological support from the LfG (for example regarding particular terms and their operationalisation in questionnaires). After the final instrument for data collection has been developed, USUMA GmbH programmes it into Voxco, a software programme for telephone interviews. In the run-up to the ad hoc survey, interviewers receive comprehensive training to familiarise themselves with the aims of the project. During these training sessions over several hours, project leaders, the LfG and the market and social research institute present the planned ad hoc survey.

A pre-test before the actual fieldwork serves to detect structural inconsistencies in data collection (for example regarding the length of questionnaires, filtering, obviously missing values regarding specific questions) and allows the survey to be fine-tuned.

In ad hoc surveys, the amount of time spent on data collection will vary depending on the research question, design and the required number of participants. In most cases, the fieldwork of ad hoc surveys will take between one and three months to complete. During the entire fieldwork, the LfG provides supervision (of interviewers) and quality assurance and thereby ensures the RKI’s standards for data collection are complied with.

**Figure 1:**
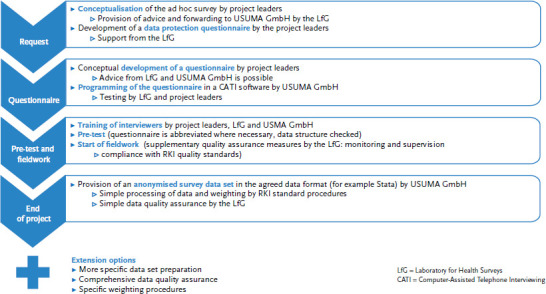
The course of the ad hoc surveys Source: Own diagram

The market and social research institute records the collected data anonymously (the following section provides further information on data protection). After finalising the fieldwork, the RKI receives the anonymised data set including a methodology report. At this point, data collection for the ad hoc survey is completed. The course of the ad hoc surveys is shown in Figure 1.

## 3. Data protection

Data protection in ad hoc surveys is based on the EU’s General Data Protection Regulation (GDPR) and a voluntary commitment to the guidelines of the ADM (Arbeitskreis Deutscher Markt- und Sozialforschungsinstitute) [10]. An important and fundamental aspect in all ad hoc surveys is ensuring that the informed consent of all interviewees has been obtained. All participants are informed at the start of the telephone interview that their participation is voluntary, receive information about the aims of the survey and data protection and are asked for their verbal consent. Further aspects of data protection measures in ad hoc surveys include:

► The separation of personal data (such as telephone numbers) and survey data is strictly observed during data collection. Personal information (in particular telephone numbers) are deleted after the survey is completed.
► Interviewers do not have access to survey data and are barred from opening closed datasets.
► Contacting interviewees, managing schedules and the interviews themselves are conducted using a specialised computer software programme. The software minimises data entry mistakes and maximises data quality. Only employees involved in process management and data analysis have access to the pool of telephone numbers of interviewees and survey data sheets.
► After finishing the ad hoc survey, USUMA GmbH provides the final data set in anonymised format to the RKI.
► Data is encoded and transferred via a cryptshare server in line with RKI provisions and in accordance with data protection regulations.

The RKI’s data protection officer ensures that each ad hoc survey complies with the generally applicable General Data Protection Regulation (GDPR) as well as Germany’s Federal Data Protection Act (BDSG-neu). To simplify the processing of the required documentation, a data protection questionnaire developed specifically for ad hoc surveys is applicable. For individual ad hoc surveys, clearance certificates from the ethics commission and the Federal Commissioner for Data Protection and Freedom of Information are obtained.

## 4. Methodology

### 4.1 Design of sampling and data collection

Ad hoc surveys are carried out as computer-assisted telephone interviewing surveys (CATI). This allows for comparisons with previous telephone interview surveys that have taken place within the context of health monitoring (GEDA 2009, GEDA 2010 or GEDA 2012) [6-8]. In principle, the same also applies to the sampling procedure. For statements on the German population in general, there are only few practicable and efficient options for sampling [11]. One method is sampling through existing official registers such as those held by official residency registries, or by using a generated telephone sampling frame. It should be noted that these concepts are both grounded on a two-step registry sample. The only difference between these sampling procedures is the selected primary survey unit (for example municipalities or types of municipalities). Another method is the ADM design for a personal face-to-face interview. As Germany, however, does not have a complete registry of all telephone numbers for personal use, the telephone sampling frame has to be generated first. The Arbeitsgemeinschaft ADM-Telefonstichproben has filled this gap by providing a sampling frame to member institutes. This generated sampling frame contains all private households in Germany that can be reached by telephone.

**Figure 2:**
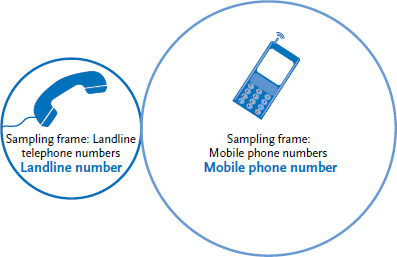
Visualisation of a dual frame sample; all telephone numbers in the sampling frame, including those that cannot be reached Source: Own diagram

For the ad hoc surveys, the ADM’s sampling system for telephone surveys, which is based on a dual frame procedure (Figure 2) is applied. The procedure consists of using two sets of numbers: the totality of mobile phone numbers and the totality of landline numbers [12].

An additional practicable dual frame for selection is the GESIS framework [13]. Except for a few minor differences (the GESIS mobile phone sample for example is based on a person-centred approach), it works in a very similar way. Figure 2 is a schematic depiction of this approach. For dual frame approaches, research currently recommends a mobile phone proportion of at least 40% [14]. Only sampling from both mobile and landline telephone number sets ensures an (almost) complete coverage of the population [15].

When a person in a household of several people is contacted, an interviewee is randomly selected. Ad hoc surveys thereby apply the Kish Selection Grid [16]. All potential interviewees in any one household are equally likely to be selected. The CATI software randomly selects the target person and the selected target person is then identified based on their recorded age and gender.

### 4.2 Call-back and sample management

The provided sample is divided into tranches, i.e. the total of telephone numbers generated is not used from the outset. The separation into tranches is aimed at minimising the number of numbers used and maximising response rates. Moreover, using tranches of telephone numbers which have not yet been used has a positive impact on the motivation of interviewers because they receive fresh numbers with every new tranche, which increases the likelihood of a successful interview [17].

Each ad hoc survey pre-defines return call rules. These rules are established based on the AAPOR standard (American Association of Public Opinion Research) [18]. Based on the ADM recommendations, the maximum number of contact attempts is limited to ten calls per number [19]. Furthermore, sample generation ensures an optimal processing of all telephone numbers and helps to achieve the highest possible response rates. Making appointments with a target person for example is awarded the highest priority because the available surveys indicate that this makes a successful interview most likely [17].

### 4.3 Pre-test

Before each ad hoc survey, a pre-test is conducted. In most cases, the pre-test is a standard pre-test in the field [20]. When testing the survey instruments, the focus is on the logical and structured use of filters, the overall design of the questionnaire (e.g. nonresponse), content related duplications and the length of the questionnaire (the time required for a successful interview). These aspects of the quality of questionnaires are evaluated based on the pre-defined parameters of programming, filter application, frequency count, spread of missing values, time required for each thematic block and feedback from interviewers and interviewees. Generally, pre-test interviews take place ten days before the main stage of the survey.

### 4.4 Fieldwork

Fieldwork here relates to data collection by telephone and all the processes associated with it, i.e. the calls made by interviewers, as well as measures of supervision and quality assurance [17]. Fieldwork is left largely in the hands of USUMA GmbH. However, LfG has a quality assurance function through monitoring the supervision and quality assurance of interviewers at their workplace.

**Figure 3:**
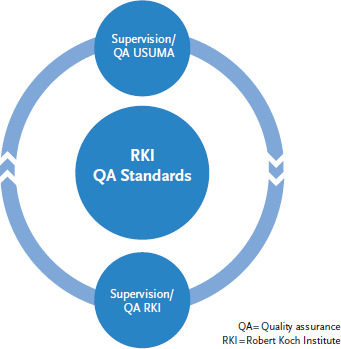
Ad hoc survey quality assurance process Source: Own diagram

The number of interviewers working on individual ad hoc surveys varies between 20 and 100. This mainly depends on the time envisaged for fieldwork and the number of interviews that have to be conducted. To minimise interviewer effects the group of interviewers should be as heterogeneous as possible [21]. For reasons of quality assurance during the fieldwork, all interviewers receive training beforehand and are thoroughly supervised during the survey. USUMA GmbH ensures the supervision of interviewers. The company thereby receives support from and is monitored by LfG (Figure 3).

The constant supervision of all interviewers serves to maintain continuous data collection quality standards. Quality assurance during the entire fieldwork is part of supervision and as such organised by LfG. Quantitative and qualitative methods aim to maintain data quality. Quantitative quality assurance includes monitoring certain process data (number of call attempts/interviews made, refusals to grant interview/telephone appointments agreed) for example. This serves chiefly to compare interviewers and identify those requiring more support as part of the qualitative measures for quality assurance being pursued. One element of qualitative methods that is used to ensure data quality during the fieldwork is the monitoring of first contact and interview situation of individual interviewers. The aim is to continuously follow-up on the first contact and interview situation of all interviewers during the fieldwork. This is achieved by using standardised quality assurance sheets, which are discussed in detail following supervision with the interviewers. Supervisors have this bank at their disposal during the entire field phase allowing them to judge the development potentials of interviewers during the fieldwork. If quality assurance reveals a poor performance on the part of individual interviewers, they are invited to participate in further training and where appropriate training in putting forward arguments [21]. Ultimately, where these measures fail to deliver results interviewers are excluded from making further calls.

### 4.5 Weighting

The ‘weighting’ of data from a random sample describes a process by which the relative importance of data from individual target persons or groups in a sample is changed. Frequently weighting factors are employed to project the results of a survey for a random sample (as opposed to the total population) in which target persons have the possibility to refuse answers. Weighting processes are differentiated between those that consider the potentially diverging probabilities with which individual target persons will select an answer (design weighting) and procedures for subsequent stratification and reducing non-response bias, i.e. bias owing to the systematic non-participation by different target population groups (calibration/post stratification). Weighting for telephone ad hoc surveys is developed in close co-operation between the RKI and the institute commissioned. Design weighting is followed by post stratification. Post stratification has primarily two goals: to increase the precision of estimate values and to reduce bias through non-response [15]. Weighting must be adapted to each specific ad hoc survey because the underlying selection processes vary, the targeted subgroups differ as does the reason for weighting. Frequent sociodemographic markers used in weighting are nevertheless age, gender, federal state and education.

### 4.6 Response rates

Response rates are an indicator of the degree to which a specific target group has been reached. They serve as a possible but not compelling quality indicator for a possible bias of the sample through the systematic non-participation of particular target groups (non-response bias). For every ad hoc survey, the response rate is calculated based on the AAPOR standard [18]. This standard is favoured particularly in survey method research and guarantees comparability with other surveys worldwide (in particular ad hoc surveys) [22]. The AAPOR standard allows the reporting of different response rates that are calculated using similar but slightly modified formulas. One of the most commonly used measures is response rate 3, RR3. This rate was calculated for GEDA 2009, GEDA 2010 and GEDA 2012, rendering comparisons with these surveys. The response rate 3 reflects the proportion of realised interviews in relation to all probable households of the total population. For those telephone numbers that remain unclear to the end of the fieldwork whether they belong to the population or not, an estimate of the proportion of those that do belong to the population is made (eligibility rate). This estimate is extracted from the data collected as a proportion of eligible valid telephone numbers (respondents and non-respondents) to all numbers with a clear status (valid and invalid telephone numbers) [23].

## 5. Discussion

The format of ad hoc surveys, as described in this article, provides the RKI with tools to quickly and efficiently generate data and related information for scientific and/or political debate. The structured processes ensure the efficient management of the respective surveys. The cornerstones are bindingly described in the framework agreement allowing for fast and transparent communication with the external service provider and ensuring that costs can be estimated beforehand.

Yet it needs to be noted that each interview format has its advantages and disadvantages. Temporal, economic, practical and methodological dimensions are all contained in the valuation standards [24]. Advantages of the telephone interview format consist of: a framework for selection, fast and up-to-date availability of data, relatively low costs, the geographic spread of interviewees, the potential to implement screening procedures (i.e. specific target popoulations) and efficient quality management of interviewers and the data collected. These advantages come up against certain disadvantages: the limited number of questions compared to surveys sent by post, the lack of possibilities for visual input to questions and a greater risk of receiving biased results due to social desirability, avoidance behaviour, or more generally through interviewer effect. Before any survey, these and other advantages and disadvantages need to be weighed up and considered within the context of the survey.

Telephone surveys, like all other forms of surveying, have experienced a continuous decrease in response rates over the last few years, even though this trend has petered off recently [25]. This development entails risks due to bias for the estimated parameters in the individual surveys. Survey research, however, indicates that low response rates do not necessarily translate into a bias of survey results (i.e. a rising non-response bias) [26].

Looking back at previous ad hoc surveys at the RKI, numerous experiences have been gained. For example it has become clear that already at the stage of questionnaire design and programming providing advice is essential because researchers have no or hardly any experience regarding the telephone interview format. This is particularly important regarding the customisation of questionnaires and related processes. Field pre-tests also revealed that the majority of implemented ad hoc surveys underestimated the true length of questionnaires. Together these experiences provide the basis for a more precise estimation of the length of questionnaires in future. Prior to the pre-testing of future ad hoc surveys the questions or indicators to be analysed using criteria (such as the number of missing values, the required response time etc.) must be clearly defined with the researchers working on questionnaire content. During the actual fieldwork, we will in future strive to further intensify the supervision of interviewers. In the past, supervision has enhanced the quality of data [21]. If the budget allows it, the aim is to increase the supervisor to interviewer ratio to one to ten, i.e. to have one supervisor for every ten interviewers. It is conceivable that qualitative quality assurance could be digitalised in the form of online feedback questionnaires.

In the long-term, the increasing need for multi-mode or mixed-mode surveys will necessitate an amplification of the framework agreement. The methodological opportunities and limitations of telephone interview surveys will therefore have to be constantly re-analysed and re-interpreted. The accumulated experience and networks of the people working at LfG will be crucial to keeping the quality of data collected at a constantly high level.

## Key statements

- The Robert Koch Institute regularly plans ad hoc telephone interview surveys and supervises their implementation.
- The Robert Koch Institute commissions an external market and social research institute with extensive experience in data collection to implement ad hoc surveys.
- One advantage to ad hoc surveys is the capacity to respond to urgent issues in a fast and flexible way.
- While based on a single methodology, depending on the research question, ad hoc surveys can be created in a bespoke way.
- After data collection, researchers at the Robert Koch Institute analyse the data.

## Figures and Tables

**Table 1:** Overview of ad hoc surveys conducted at the Robert Koch Institute Source: Own table

Short name	Title	Survey period	Net number of participants (n)
KomPaS	Survey on Communication and Patient-safety	12.06.2017-22.09.2017	5,053
Diabetes	Disease knowledge and information needs - Diabetes mellitus	23.08.2017-30.11.2017	3,807 total (of these 1,479 people with diabetes)
Salmonella Kottbus	Case control study of an outbreak of Salmonella Kottbus	15.08.2017-19.08.2017	96
Listeriosis	Case control study of an outbreak of Listeriosis	22.08.2017-26.08.2017	28
Nutrition Survey	Nutrition survey	25.09.2017-14.11.2017	1,010
TAMIA	Telephone Survey on Vaccine Hesitancy among Family Physicians in Germany	08.11.2017-10.01.2018	701
IMIRA-Befragungsstudie	Improving Health Monitoring in Migrant Populations - IMIRA Survey	15.01.2018-31.05.2018	1,190
